# β-Hydroxybutyrate Attenuates Cardiac Inflammation and Hepatic Fibrosis in Dahl Salt-Sensitive Rats

**DOI:** 10.3390/cimb48060620

**Published:** 2026-06-16

**Authors:** Satoyasu Ito, Eri Manabe, Toshiyuki Shikata, Kojiro Takamoto, Shuhei Kobuchi

**Affiliations:** 1 Division of Clinical Pharmacy, Department of Pharmacy, School of Pharmacy, Hyogo Medical University, Kobe 650-8530, Japan; 2 Department of Cardiovascular and Renal Medicine, School of Medicine, Hyogo Medical University, Nishinomiya 663-8501, Japan; 3 Department of Pharmacy, Hyogo Medical University Hospital, Nishinomiya 663-8501, Japan; 4 Division of Pharmacology, Department of Pharmacy, School of Pharmacy, Hyogo Medical University, Kobe 650-8530, Japan

**Keywords:** hypertension, β-hydroxybutyrate, fibrosis, inflammation, liver

## Abstract

Hypertension remains a major driver of multi-organ damage, including cardiac remodeling and hepatic complications. The ketone body β-hydroxybutyrate (BHB) has emerged as a potential metabolic signaling molecule with anti-inflammatory properties. This study investigated whether BHB attenuates cardiac stress and hepatic injury in a salt-sensitive hypertensive model. Dahl salt-sensitive (DS) rats were fed a high-salt (HS) diet combined with a choline-deficient diet to induce cardiac inflammation and hepatic fibrosis. Rats received either BHB or a control vehicle. We found that BHB significantly suppressed hepatic lipid accumulation and fibrotic markers, including TGF-β and collagen III mRNA, even under severe dietary stress. In the heart, BHB attenuated the expression of inflammatory markers (TNF-α and ANP) despite the persistence of high systolic blood pressure. These results demonstrate that BHB exerts direct organ-protective effects through anti-inflammatory and anti-fibrotic actions that are independent of robust blood pressure reduction. Our findings suggest that BHB could be a promising metabolic intervention for managing multi-organ complications in hypertensive patients with metabolic comorbidities.

## 1. Introduction

Hypertension is a major global health problem and a leading risk factor for cardiovascular morbidity and mortality [1,2]. Persistent elevation of blood pressure induces structural and functional alterations in multiple organs, particularly the heart, leading to cardiac remodeling and dysfunction [2,3]. In addition to cardiovascular complications, hypertension is increasingly recognized as a systemic disorder associated with metabolic disturbances affecting extra-cardiac organs, including the liver [4,5].

The Dahl salt-sensitive rat is a well-established experimental model of salt-sensitive hypertension and has been widely used to investigate mechanisms of hypertension-induced organ damage [6,7]. In this model, sustained pressure overload leads to cardiac hypertrophy and inflammatory activation, both of which contribute to disease progression [8].

Accumulating evidence indicates that inflammation plays a central role in hypertension-related organ damage [9,10]. In the heart, hypertensive stress increases the expression of atrial natriuretic peptide (ANP) and pro-inflammatory cytokines such as tumor necrosis factor-α (TNF-α), which contribute to cardiac remodeling and dysfunction [11,12]. Emerging evidence also suggests that metabolic remodeling contributes to cardiac dysfunction, particularly in conditions such as heart failure with preserved ejection fraction [13].

In parallel, metabolic and inflammatory stress associated with hypertension may also affect the liver, promoting hepatic steatosis and fibrosis [11,12,13]. These changes are characterized by lipid accumulation and activation of fibrogenic pathways, including transforming growth factor-β (TGF-β) signaling. Recent studies have further highlighted the systemic interactions between hepatic dysfunction and cardiovascular disease [4,5].

Ketone bodies, particularly β-hydroxybutyrate (BHB), have recently attracted attention as metabolic regulators with pleiotropic biological effects [14,15]. Beyond their role as alternative energy substrates, BHB exerts anti-inflammatory and antioxidative effects and has been implicated in cardiovascular protection [16,17]. In particular, BHB has been reported to inhibit inflammatory signaling pathways, including activation of the NLRP3 inflammasome [17], suggesting a potential protective role in cardiovascular and metabolic diseases. These metabolic adaptations are closely linked to myocardial energy utilization and mitochondrial function [18,19]. Recent studies have further highlighted the role of ketone bodies as important metabolic and signaling molecules in cardiovascular and metabolic diseases [20,21,22,23,24].

Despite these findings, the effects of BHB on hypertension-induced cardiac and hepatic injury remain incompletely understood. In particular, whether BHB simultaneously attenuates cardiac stress, hepatic steatosis, and fibrosis independently of blood pressure changes, has not been fully elucidated.

Therefore, in the present study, we investigated the effects of BHB on blood pressure, cardiac stress, and hepatic lipid accumulation and fibrosis in Dahl salt-sensitive rats. We hypothesized that BHB attenuates hypertension-associated organ damage through anti-inflammatory and metabolic regulatory mechanisms, independent of robust blood pressure reduction. While several studies have explored the metabolic role of BHB, its direct effects on the cardio-hepatic axis under severe salt-sensitive hypertension remain to be fully elucidated. This study serves as an initial molecular characterization of BHB’s protective potential in this complex comorbid model.

## 2. Materials and Methods

### 2.1. Animals and Experimental Design

For all experiments, male DS rats (DIS/EisSlc, formerly named Dahl/SS-Iwai S) were purchased from Japan SLC, Inc. (Shizuoka, Japan). Rats were housed in stainless steel cages (KN-601, Natsume Seisakusho Co., Ltd., Tokyo, Japan) with wooden bedding (White flakes, The Jackson Laboratory Japan, Inc., Tokyo, Japan) in a light-controlled room under a 12 h light/dark cycle, were kept in the environment at a temperature of 23 ± 3 °C and a humidity level of 50% ± 10%, and had free access to food and water. Five-week-old rats were fed a normal salt diet (0.3% NaCl; Oriental Yeast Co., Ltd., Tokyo, Japan) and allowed to acclimatize for a week.

At 6 weeks of age, they were randomly assigned to five groups:

(1) Control,
(2) HS (choline-supplemented amino acid-defined diet),
(3) HS + BHB,
(4) HS + CD (choline-deficient amino acid-defined diet), and
(5) HS + CD + BHB.

At 13 weeks of age, rats were deeply anesthetized with an intraperitoneal injection of medetomidine hydrochloride (0.15 mg/kg), midazolam (2 mg/kg), and butorphanol tartrate (2.5 mg/kg) and euthanized by exsanguination via bilateral common carotid artery transection. Blood and organs were collected for subsequent analyses. Each group consisted of *n* = 12 animals. Body weight differed significantly among groups (one-way ANOVA, *p* < 0.0001). Importantly, body weight did not correlate with the primary outcome measures, indicating that the group differences in body weight did not influence the main findings.

### 2.2. Diets and Induction of Liver Fibrosis

To induce hepatic fibrosis, a choline-deficient amino acid-defined diet (no-choline diet) was used as previously described [25]. A choline-supplemented amino acid-defined diet (amino diet) was used as the corresponding control diet.

All experimental groups (HS, HS + BHB, HS + CD, and HS + CD + BHB) were fed a high-salt diet containing 8% NaCl to induce salt-sensitive hypertension. Accordingly, the “HS” designation in group names reflects this uniform high-salt loading applied to all non-Control groups. The Control group alone was maintained on a normal-salt diet (0.3% NaCl).

The CDAA control diet contained amino acids as the protein source, L-methionine (5.1 g/kg diet), and choline bitartrate (2.5 g/kg diet). The fibrogenic CDAA diet was identical except that methionine content was reduced (1.7 g/kg diet) and choline was omitted.

### 2.3. D-β-Hydroxybutyrate Supplementation

D-β-hydroxybutyric acid (BHB, OKETOA^®^) was diluted to 20% in drinking water and given ad libitum to the amino HS + BHB group and the no-choline HS + CD + BHB group (Osaka Gas Chemicals Co., Ltd., Osaka, Japan).

### 2.4. Blood Pressure Measurement

Systolic blood pressure was measured weekly by the tail-cuff method (BP-98A, Softron, Tokyo, Japan) in conscious, prewarmed rats. Measurements were averaged from at least five consecutive recordings per session.

### 2.5. Sample Collection

At the end of the feeding period, rats were anesthetized, and blood was collected via cardiac puncture. Liver, heart, kidneys, and spleen were excised, weighed, and processed for molecular and histological analyses. The left ventricle was separated from the right ventricle and septum for molecular analyses.

### 2.6. Biochemical Measurements

Serum ketone bodies were measured using β-Hydroxybutyrate (Ketone Body) Colorimetric Assay Kit (Cayman Chemical, Ann Arbor, MI, USA). Serum levels of creatinine were analyzed by Lab Assay Creatinine (Fujifilm Wako Chemicals Corporation, Osaka, Japan).

### 2.7. Quantification of mRNA

Total RNA was extracted from the heart and liver using TRIzol reagent (Thermo Fisher Scientific, Tokyo, Japan). Total RNA was reverse transcribed into cDNA by the High-Capacity cDNA Reverse Transcription Kit (Thermo Fisher Scientific, Tokyo, Japan) using the GeneAmp PCR System 9700 (Thermo Fisher Scientific, Tokyo, Japan). Real-time polymerase chain reaction quantification of transcripts was performed using the TaqMan Gene Expression Master Mix and TaqMan Gene Expression Assays (Applied Biosystems, Foster City, CA, USA) using the StepOnePlus Real-Time PCR System (Thermo Fisher Scientific, Tokyo, Japan). TaqMan Gene Expression Assays of natriuretic peptide A (Nppa: Rn00664637_g1), collagen (col3a1: Rn01437681_m1) and transforming growth factor (TGF)-β 1 (Tgfb1: Rn22572010_m1) were used.

### 2.8. Histological Analysis

For Masson’s trichrome staining, liver tissue was fixed with formalin and embedded in paraffin, and sections were prepared for histological analysis. Liver fibrosis was quantitatively analyzed in the stained area using image analysis software. For lipid accumulation, which was evaluated using Oil Red O staining, the tissue was rapidly frozen in embedding material (O.C.T. compound: Sakura Finetek Japan Co., Ltd., Tokyo, Japan), and frozen sections were prepared using a cryostat.

### 2.9. Statistical Analysis

All data were analyzed using EZR (Saitama Medical Center, Jichi Medical University, Saitama, Japan). Statistical analyses were performed on individual raw values. Normality was assessed using the Shapiro–Wilk test before applying parametric analysis. As all datasets satisfied normality assumptions, comparisons among multiple groups were conducted using one-way analysis of variance (ANOVA) followed by Tukey’s multiple comparison test. Although data are presented as box-and-whisker plots (median, interquartile range, and minimum–maximum values) to illustrate data distribution, statistical comparisons were performed using parametric testing on raw data. A value of *p* < 0.05 was considered statistically significant.

### 2.10. Ethical Approval

All experimental procedures were approved by the Animal Care and Use Committee of Hyogo Medical University and conformed to the Guidelines for Proper Conduct of Animal Experiments by the Science Council of Japan (23-108A).

## 3. Results

### 3.1. Effects of BHB on Blood Pressure

**Figure 1:**
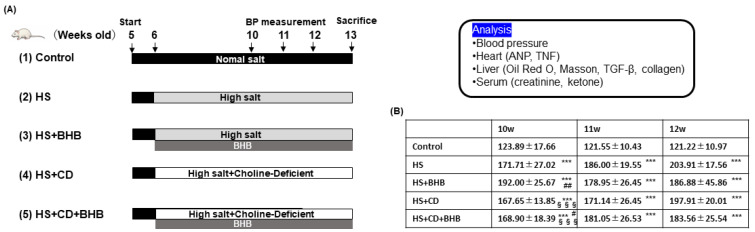
Experimental design and blood pressure analysis. (**A**) Schematic representation of the experimental protocol. Dahl salt-sensitive rats were assigned to five groups: control, HS, HS + BHB, HS + CD, and HS + CD + BHB. BHB was administered as 20% in drinking water. Blood pressure was measured at 10, 11 and 12 weeks of age, and animals were sacrificed at 13 weeks for tissue and serum analyses. (**B**) Systolic blood pressure in each group at 10, 11 and 12 weeks of age. Data are presented as mean ± standard deviation (SD). Statistical comparisons were performed using one-way ANOVA followed by Tukey’s multiple comparison test. *** *p* < 0.001 vs. Control; # *p* < 0.05, ## *p* < 0.01 vs. HS; §§§ *p* < 0.001 vs. HS + BHB (Tukey’s post hoc test).

Systolic blood pressure differed significantly among groups at all time points (10, 11, and 12 weeks; one-way ANOVA, *p* < 0.001 for all; Figure 1B).

At 10 weeks, systolic blood pressure was significantly higher in all experimental groups compared with the control group (*p* < 0.001 for all comparisons). Notably, systolic blood pressure was significantly higher in the amino HS+ BHB group than in the HS group (*p* < 0.01).

At 11 weeks, systolic blood pressure remained significantly elevated in all experimental groups compared with the control group (*p* < 0.001). However, no significant differences were observed among the HS, HS + BHB, HS + CD, and HS + CD + BHB groups.

At 12 weeks, systolic blood pressure was markedly increased in all experimental groups compared with the control group (*p* < 0.001). No significant differences were observed among the experimental groups, although a trend toward lower blood pressure was observed in BHB-treated groups.

### 3.2. Effects of BHB on Cardiac Stress, Inflammation, and Hypertrophy

**Figure 2:**
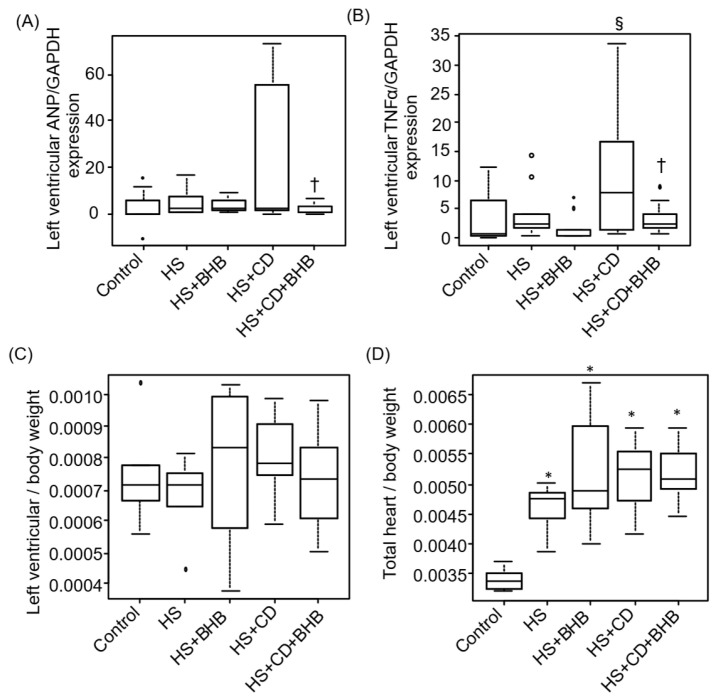
Cardiac gene expression analysis and cardiac weight parameters. (**A**) Left ventricular atrial natriuretic peptide (ANP) mRNA expression. (**B**) Left ventricular tumor necrosis factor-α (TNF-α) mRNA expression. (**C**) Left ventricular weight normalized to body weight. (**D**) Total heart weight normalized to body weight. Relative mRNA expression levels of cardiac hypertrophy and inflammation markers, including atrial natriuretic peptide (ANP) and tumor necrosis factor-α (TNF-α), in each group. Gene expression levels were normalized to GAPDH and expressed relative to the control group. Data are presented as box-and-whisker plots (median, interquartile range, and range) with individual data points. (**A**) ANP expression tended to be elevated in the HS + CD groups, while BHB treatment suppressed these increases compared with the HS + CD group. (**B**) The HS + CD group was elevated in TNF-α expression levels. BHB administration suppressed these increases. Statistical analysis was performed using one-way ANOVA. *p* < 0.05 was considered statistically significant. § *p* < 0.05 vs HS + BHB; † *p* < 0.05 vs HS + CD (Tukey’s post hoc test). * *p* < 0.05 vs. Control; § *p* < 0.05 vs. HS + BHB; † *p* < 0.05 vs. HS + CD.

Cardiac stress and inflammatory markers are shown in Figure 2A,B. Left ventricular ANP expression was significantly increased in the no-choline group compared with the control group and was reduced by BHB treatment. Similarly, TNF-α expression was elevated in the no-choline group and attenuated in the no-choline + BHB group.

Cardiac hypertrophy parameters are shown in Figure 2C,D. Left ventricular weight normalized to body weight did not differ significantly among groups. In contrast, total heart weight normalized to body weight was significantly increased in the amino and no-choline groups compared with the control group. BHB treatment did not significantly affect these parameters.

### 3.3. Effects of BHB on Hepatic Lipid Accumulation and Fibrosis

**Figure 3:**
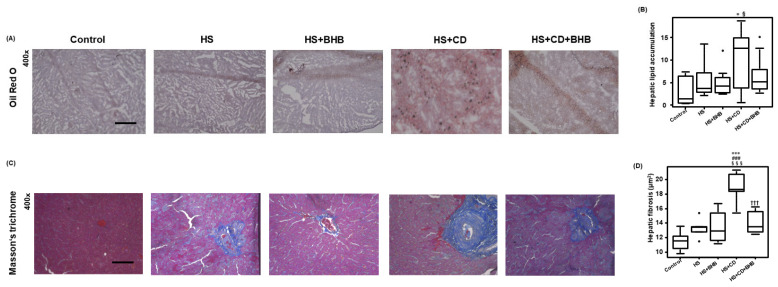
Hepatic lipid accumulation and fibrosis. (**A**,**B**) Representative images and quantitative analysis of Oil Red O staining in liver sections from each group. Lipid accumulation was markedly increased in the HS + CD group and moderately increased in the HS group compared with the control group. BHB treatment reduced hepatic lipid accumulation in both groups. (**C**,**D**) Representative images and quantitative analysis of Masson’s trichrome staining. Fibrotic areas were significantly increased in the no-choline group compared with the control group, whereas BHB treatment attenuated hepatic fibrosis. Quantitative data are presented as box-and-whisker plots (median, interquartile range, and min–max). Scale bars = 50 μm (original magnification ×400). Statistical analysis was performed using one-way ANOVA followed by Tukey’s multiple comparison test. *p* < 0.05 was considered statistically significant. * *p* < 0.05, *** *p* < 0.001 vs. Control; ### *p* < 0.001 vs. HS; § *p* < 0.05; §§§ *p* < 0.001 vs. HS + BHB; ††† *p* < 0.001 vs. HS + CD.

Hepatic lipid accumulation and fibrosis are shown in Figure 3. Oil Red O staining demonstrated increased lipid accumulation in both the HS and HS + CD groups, with the highest levels observed in the HS + CD group. BHB treatment reduced hepatic lipid accumulation in both dietary conditions.

Masson’s trichrome staining revealed marked hepatic fibrosis in the HS + CD group compared with the control group. Quantitative analysis confirmed a significant increase in fibrotic area in the HS + CD group, which was significantly attenuated by BHB treatment.

### 3.4. Effects of BHB on Hepatic Fibrosis-Related Gene Expression

**Figure 4:**
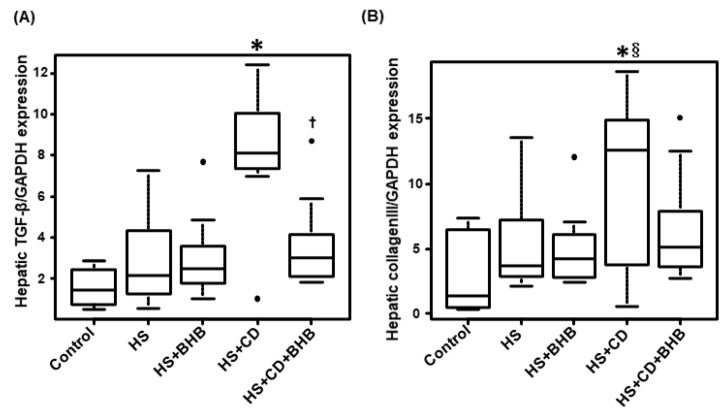
Hepatic fibrosis-related gene expression. Relative mRNA expression levels of fibrosis-related markers, including transforming growth factor-β (TGF-β) and collagen III, in liver tissues. (**A**) TGF-β expression differed significantly among groups (one-way ANOVA, *p* < 0.001). TGF-β expression was markedly increased in the HS + CD group compared with the control group and was significantly attenuated by BHB treatment. Expression levels in the HS and HS + BHB groups were moderately elevated compared with control. (**B**) Collagen III expression also differed significantly among groups (one-way ANOVA, *p* < 0.001). Collagen III expression was significantly increased in the HS + CD group compared with the control and HS + BHB groups and was partially reduced by BHB treatment. Gene expression levels were normalized to GAPDH and expressed relative to the control group. Data are presented as box-and-whisker plots (median, interquartile range, and minimum–maximum values). Statistical comparisons were performed using one-way ANOVA followed by Tukey’s multiple comparison test. * *p* < 0.05 vs. control; § *p* < 0.05 vs. HS + BHB; † *p* < 0.05 vs. HS + CD.

Hepatic fibrosis-related gene expression is shown in Figure 4. TGF-β mRNA expression differed significantly among groups (one-way ANOVA, *p* < 0.001). TGF-β expression was markedly increased in the HS + CD group compared with the control group and was significantly reduced by BHB treatment.

Collagen III mRNA expression also differed significantly among groups. Collagen III expression was significantly elevated in the HS + CD group compared with the control group. BHB treatment partially attenuated this increase, although the reduction was less pronounced than that observed for TGF-β expression.

### 3.5. Effects of BHB on Serum Biochemical Parameters

**Figure 5:**
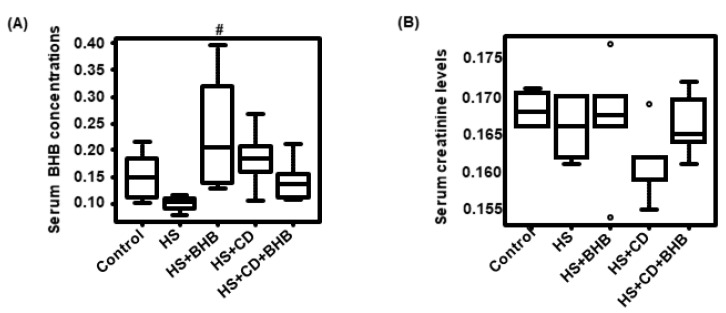
Serum biochemical parameters. Serum creatinine levels and circulating ketone body concentrations. (**A**) No significant differences in serum creatinine levels were observed among groups. BHB levels were significantly higher in the HS + BHB group compared with the HS group, confirming effective BHB administration. Data are presented as box-and-whisker plots (median, interquartile range, and range). Statistical comparisons were performed using one-way ANOVA (*p* < 0.05) followed by Tukey’s multiple comparison test. # *p* < 0.05 vs. HS group. (**B**) Serum creatinine levels did not differ significantly among groups, indicating preserved renal function under the experimental conditions.

Serum biochemical parameters are shown in Figure 5. Circulating BHB levels differed among groups, with significant elevation observed in the HS + BHB group compared with the HS group. In contrast, no significant increase was observed in the HS + CD + BHB group.

## 4. Discussion

A key observation of the present study is that BHB attenuated cardiac inflammatory and stress markers despite the persistence of severe systolic hypertension. While the exact pathways were not directly quantified in this animal cohort, several distinct molecular mechanisms likely explain these blood pressure-independent cardioprotective effects. First, BHB is a well-known endogenous inhibitor of class I histone deacetylases (HDACs), particularly HDAC1, HDAC3, and HDAC4. Previous studies have demonstrated that HDAC inhibition by BHB can epigenetically suppress the transcription of pro-inflammatory cytokines, including TNF-α, and blunt hypertrophic gene programs in cardiomyocytes even under conditions of mechanical stretch and pressure overload [17,21]. Second, BHB acts as a specific agonist for the hydroxycarboxylic acid receptor 2 (HCA2), which is highly expressed on immune cells such as macrophages and monocytes. Activation of HCA2 has been shown to induce an anti-inflammatory phenotype, suppressing systemic and localized inflammatory cascades, which subsequently mitigates secondary myocardial stress and drops ANP signaling [20,23]. Third, under severe hypertensive stress, the myocardium undergoes a metabolic shift away from fatty acid oxidation. Under these conditions, BHB serves as a highly oxygen-efficient energetic substrate, providing more ATP per mole of oxygen consumed compared to fatty acids. This metabolic optimization enhances myocardial energetic efficiency, directly reducing localized cellular distress and lowering ANP expression without requiring a reduction in peripheral vascular resistance or systemic blood pressure [13,16].

Consistent with these theoretical mechanisms, our results showed that BHB suppressed the expression of specific markers of myocardial stress (ANP) and inflammation (TNF-α) (Figure 2). These findings suggest that BHB exerts organ-protective effects, at least in part, through the localized modulation of inflammatory and stress pathways. Hypertension is closely associated with inflammation-mediated organ injury [9,10]. In the present study, increased myocardial TNF-α expression in the HS + CD group was attenuated by BHB treatment, supporting the hypothesis that the anti-inflammatory effects of BHB contribute to protection against localized tissue injury. In addition, the reduction in ANP expression indicates that BHB successfully alleviates hemodynamic-induced cellular distress under hypertensive conditions.

However, caution must be exercised when interpreting these molecular changes as definitive evidence of long-term functional cardioprotection. Ventricular filling pressures, congestion, and overall myocardial performance were not directly assessed in the present study. Furthermore, the classical concept of a direct continuum from hypertensive hypertrophy to intrinsic myocardial failure remains highly debated. As recently highlighted by Bellicini (2026), a substantial proportion of the excess cardiovascular risk in sustained hypertension is mediated through coronary artery disease and atherosclerotic complications, rather than through an inherent progression of hypertensive remodeling toward isolated myocardial decompensation [26]. Therefore, the BHB-mediated reductions in ANP and TNF-α observed in our model most likely represent a mitigation of acute localized myocardial stress and inflammatory signaling, contributing to overall cardiovascular risk reduction rather than the direct arrest of an intrinsic cardiomyopathy substrate.

In the liver, lipid accumulation and fibrotic changes were markedly increased in the HS + CD group, as demonstrated by Oil Red O and Masson’s trichrome staining (Figure 3). These pathological alterations were accompanied by increased transcriptional expression of TGF-β and collagen III, key drivers of fibrogenesis. BHB treatment significantly attenuated these alterations, suggesting that BHB exerts robust protective effects against hepatic steatosis and fibrosis even under severe dietary and hypertensive stress. The marked increase in hepatic TGF-β expression in the HS + CD group and its reduction by BHB further support this direct antifibrotic capability.

Interestingly, we observed an intriguing discrepancy in circulating ketone bioavailability: while BHB supplementation successfully elevated steady-state serum BHB levels in the healthy liver condition (HS + BHB group), it did not cause a statistically significant elevation in the diseased condition (HS + CD + BHB group). This phenomenon can be explained by altered metabolic handling under multi-organ stress. First, the severe hepatic steatosis and advanced fibrosis induced by the choline-deficient diet may directly impair the gastrointestinal absorption, portal transport, or metabolic processing of exogenously administered BHB. Second, and perhaps more importantly, under the dual stress of severe salt-sensitive hypertension and metabolic disruption, peripheral tissues—most notably the pressure-overloaded, hypertrophied heart—drastically accelerate their uptake and utilization (ketolysis) of BHB as an emergency fuel source [13,15]. Consequently, the exogenously supplied BHB may have been rapidly cleared from the circulation by the distressed myocardium to sustain energetic demands, preventing an increase in resting serum concentration while successfully driving localized anti-inflammatory and stress-reducing actions within the target organs.

Importantly, serum creatinine levels were not significantly different among groups, indicating that renal function was preserved under the experimental conditions (Figure 5). These findings suggest that BHB does not exert adverse effects on renal function in this comorbid model.

Several limitations should be acknowledged. First, the sample size was relatively small, which may have limited statistical power for secondary parameters. Second, the effects of dietary interventions without high-salt loading were not evaluated, as the primary focus was on the rescue effects of BHB under severe hypertensive stress. Third, this study was conducted in an animal model, and direct translation to human clinical hypertension requires caution. Most importantly, the anti-inflammatory and anti-fibrotic actions of BHB in this study were demonstrated primarily through quantitative mRNA expression profiling combined with definitive histological phenotypes. We found a highly consistent correlation between the transcriptional downregulation of fibrogenic/inflammatory genes and the structural rescue of tissue architecture (e.g., reduced fibrotic area and hepatic lipid accumulation). Nevertheless, the lack of direct protein-level validation (such as Western blotting or ELISA) for specific downstream signaling cascades, such as the NLRP3 inflammasome, remains a distinct limitation of this initial characterization. Because specific intracellular pathway blockades or protein-level kinetics were not executed, our findings regarding the precise molecular switches remain associative. Future investigations utilizing Western blotting, immunohistochemistry, and pathway-specific knockout models are warranted to definitively establish the causal protein networks driving BHB-mediated cardiorenal and hepatic protection.

## 5. Conclusions

BHB attenuates severe dietary- and salt-induced cardiac stress signaling and hepatic injury in Dahl salt-sensitive rats, although its systemic bioavailability and effects depend on the prevailing metabolic and organ-pathological context. These findings suggest that BHB represents a promising metabolic intervention targeting multi-organ complications in hypertensive patients with metabolic comorbidities.

## Data Availability

The original contributions presented in this study are included in the article. Further inquiries can be directed to the corresponding author.
